# Financialisation, regional economic development and the coronavirus crisis: a time for spatial monetary policy?

**DOI:** 10.1093/cjres/rsab033

**Published:** 2021-11-14

**Authors:** Martin Sokol, Leonardo Pataccini

**Affiliations:** 1 Department of Geography, Trinity College Dublin, Dublin, Ireland; 2 Department of Global Economics Interdisciplinary Studies, University of Latvia, Riga, Latvia

**Keywords:** financialisation, crisis, central banks, monetary policy, spatial policy, regional development

## Abstract

This paper argues that ‘spatial monetary policy’ may be needed to achieve more territorially balanced economic development. Central banks have been key in fostering financialised economies while also preventing their collapse in times of crisis—a role further strengthened by the coronavirus pandemic. Central banks have thus become the most powerful economic policy-making institutions, just when spatial disparities are likely to deepen. In the context of crisis-ridden financialised capitalism, regional development policies should consider the spatial implications of central bank interventions and recognise monetary policy as a key element of spatial policy. Simultaneously, monetary policy should embrace an explicit spatial agenda.

## Introduction

This paper argues that in order for spatial policies to achieve more territorially balanced economic development, they need to form an integral part of the most influential economic policy of crisis-ridden financialised capitalism, namely monetary policy. Until now, with few exceptions, monetary policy has been largely absent from debates on urban and regional development (see also Martin, 2015). This is a major blind spot of spatial policies. We argue that a frequent preoccupation with themes such as regional innovation (valuable as this is) should not distract from macro-economic forces and processes that have strong bearings on local and regional economic fortunes. An obvious point to make here is that advanced capitalist economies have been, in the last few decades, fundamentally transformed by the process of financialisation (for example, Epstein, 2005; Krippner, 2005; van der Zwan, 2014). Financialisation, or the growing power of finance in the economy, has been defined by Aalbers (2016, 2) as ‘the increasing dominance of financial actors, markets, practices, measurements and narratives, at various scales, resulting in a structural transformation of economies, firms (including financial institutions), states and households’. The manifestations of these structural transformations include (but are not limited to) the growing weight of finance in the economy relative to production; a growing reliance of firms on market-based finance (shadow banking) relative to bank-based finance; a reorientation of bank lending toward households; and the growing debt of households including mortgage debt (for example, see Aalbers, 2016; Braun and Gabor, 2020; Lapavitsas et al., 2012, 1–2; Stockhammer, 2008, 2012). Financialisation has changed the dynamics of economic processes—often with adverse consequences for many regions (Pike, 2006; Pike and Pollard, 2010; see also Schwan, 2017; Sokol, 2013), for entire national economies (for example, Celi et al., 2018; Lapavitsas et al., 2012) and for the stability of the system as a whole (for example, Aalbers, 2016; Celi et al., 2018; Lapavitsas et al., 2012; Stockhammer, 2012).

What is critical to recognise though, is that central banks have played a central role in promoting financialisation and the accompanying shift in gravity away from the productive economy and towards the financial sphere. The actions of central banks—the US Federal Reserve (the Fed), the European Central Bank (ECB), the Bank of England and the Bank of Japan among others—have also been decisive in preventing a systemic collapse in times of crisis. As a consequence of these developments, central banks have, in the context of financialisation, became the most powerful economic policy-making institutions in contemporary, crisis-ridden capitalism (see also Braun, 2020a; Jackson, 2020a; Tooze, 2020a).

We argue that, hand in hand with the increasing power of central banks, the spatial effects of monetary interventions have become ever more significant. The role of central banks (and the spatial implications of monetary policies) will further increase due to the pandemic-induced crisis—precisely at the moment when spatial divides and regional disparities are likely to deepen. A progressive agenda for regional development in the 21st century should recognise central banks as key economic policy-making institutions with significant spatial implications, and monetary policy as a key element of spatial policy. Simultaneously, monetary policy should embrace an explicit spatial agenda. In short, ‘spatial monetary policy’ is needed. Such ‘spatial monetary policy’ must work in concert with fiscal policy in order to achieve more territorially balanced development.

The remainder of the paper is organised as follows. Section 2 provides a brief overview of literatures situated at the intersections between monetary theories, spatial theories and studies of financialisation. Section 3 will turn to examine the practice of central banking and monetary policies in the era of financialisation. Section 4 will highlight the case of the ECB and the spatial-monetary challenges of the eurozone. Section 5 will argue that the coronavirus pandemic offers an opportunity for central banks to introduce a ‘spatial monetary policy’. Finally, Section 6 will summarise the main arguments.

## Theoretical perspectives: monetary theories, spatial theories and financialisation studies

**Figure 1.**
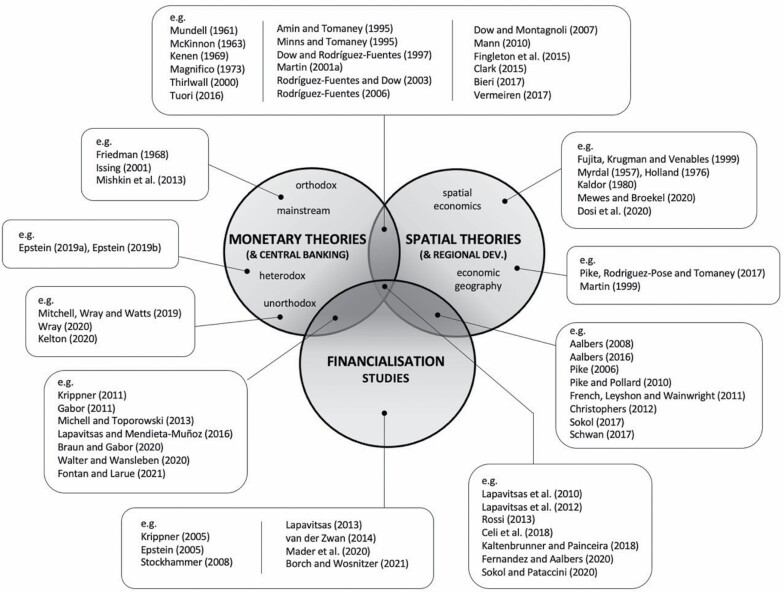
Monetary theories, spatial theories and financialisation studies. Source: Authors.

As already mentioned above, monetary policy has been largely absent from spatial policy and regional development debates, with few exceptions (for example, Amin and Tomaney, 1995; Fingleton et al., 2015; Martin, 2001a; Minns and Tomaney, 1995). Equally, spatial considerations are seldom part of the debates on monetary policy. Yet, space clearly matters to monetary arrangements. Likewise, monetary arrangements matter a great deal for spatial development. The emergence of financialisation adds a further dimension to these considerations; financialisation affects, and is affected by, both monetary arrangements and spatial structures. This section aims to highlight some of these connections by providing a brief overview of key literature, focusing on intersections (overlaps) between monetary theories, spatial theories and financialisation studies (see Figure 1).

Figure 1 is, of course, a simplification of what is a complex and evolving body of knowledge(s). Indeed, it is important to note that each of the three fields (circles in Figure 1) represents a diverse set of literatures, often with very different conceptualisations of key issues. The literature on *monetary theories* (and theories of central banking), for instance, includes a range of approaches, from orthodox to mainstream to heterodox and unorthodox. These approaches offer competing, and often contradictory, accounts of monetary issues. As Dow (2020, 59) has noticed, for example, the Neo-Austrian approach (of the Hayekian tradition) ‘explicitly advocated the privatisation of money’, reliance on market forces and ‘as little government involvement in finance as possible’. In such a world, there would ‘no longer be any need for a central bank’ (Dow, 2020, 60). In contrast, the proponents of the Modern Monetary Theory (see Section 5) consider central banks as being absolutely crucial for making contemporary economies work.

In a similar vein, the vast body of literature on *spatial theories* (and theories of regional development) is a conglomerate of very diverse approaches (for example, see Pike et al., 2017). This ranges from approaches that are deeply anchored in economics and could best be described as ‘spatial economics’—including the Krugman-style ‘New Economic Geography’ (for example, Fujita et al., 1999)—to new economic geography within ‘economic geography proper’ (see Martin, 1999). What is important to highlight here is that even approaches that share a common disciplinary background often provide contradictory conclusions to the most fundamental issues. Of particular interest here is, of course, the key question of whether economic development trajectories of regions within a market economy will converge or diverge. This ‘simple’ question dominates much of the debate in the field—from the classic works of Myrdal, (1957), Holland (1976) and Kaldor (1980) to the most recent contributions such as Mewes and Broekel (2020) and Dosi et al. (2020)—and becomes even more critical when considered together with monetary aspects.

Unfortunately, monetary theories and theories of central banking (for example, Friedman, 1968; Issing, 2001) do not usually consider space and geography as crucial to their theorisation. Likewise, spatial theories and theories of regional economic development do not necessarily see central banking and monetary structures as being crucial to their conceptualisation of spatial processes. However, neither of the two fields can afford to ignore the other. Indeed, an important body of work has emerged over time at the intersection of monetary and spatial theories (Figure 1). This body of work highlights the fact that space matters to monetary structures and vice-versa. But how exactly space and monetary structures matter to each other is the subject of considerable debate.

There is, for instance, a major debate around Mundell’s (1961) concept of the ‘optimum currency areas’ (OCAs), see Martin (2001a, 54–58). The issue of an optimal area for a currency (monetary) union is not only about its geographical extent (that is, how big an area it should cover), but also about its internal economic geography: to what degree should constituent parts (regions or whole nations) be homogeneous for a monetary union to work? Four factors (homogeneity conditions) are usually emphasised: economic similarity (for example, similar levels of economic openness/trade; similar degrees of structural diversification); mobility of factors of production (the ease with which capital and labour can move around); similar propensity to inflation; and the existence of automatic fiscal stabilisation mechanisms (Kenen, 1969; Magnifico, 1973; McKinnon, 1963). It is not entirely clear, however, exactly how much these conditions matter, or what happens if these conditions are not met (see Martin, 2001a, for a good discussion).

A key economic rationale for forming a monetary union is of course the expectation that its constituent parts are better off together than apart. However, in a real-world situation, it is often hard to show that this is the case—not least because of a missing counterfactual: what would happen without the given monetary union? Equally problematic (but equally important) is the following related question: do individual constituent parts of a monetary union converge or diverge in their development trajectories? In other words, do poorer regions (or whole nations) catch up with the richer ones, or do they fall further behind? Answers to such questions are crucial for the European project of the Economic and Monetary Union (EMU), for example, where nation-states give up important parts of their economic sovereignty for the ‘greater good’ (see also Section 4). Opinions on this matter are split; some argue that a monetary union should not be forced on economies that are economically heterogeneous in the first place, while others argue that the very emergence of a monetary union will accelerate the process of homogenisation needed for its effective functioning. In contrast to such optimistic (convergence) views (for example, Gill and Raiser, 2012), sceptics argue that a monetary union will lead to divergence, that is, it will further deepen economic differences between its constituent parts (Thirlwall, 2000; see also Celi et al., 2018). It may also be the case that there is ‘no clear or definitive answer to these questions’ yet (Martin, 2001a, 74). It is, of course, possible that both convergence and divergence processes are in operation simultaneously (cf. Myrdal, 1957) or that the dominance of either convergence or divergence can change over time. Further to this, it is important to note that it is not just being part of a monetary union that matters, but also what kind of monetary policies are implemented by the central bank (see Section 3).

We would like to argue that the matter is further complicated by the onset of financialisation (for example, Borch and Wosnitzer, 2021; Epstein, 2005; Mader et al., 2020; Krippner, 2005; Stockhammer, 2008; van der Zwan, 2014). Financialisation has major implications for the entire financial sphere, including central banking, and there is a growing body of literature at the intersection of *monetary theories* and *financialisation studies* (Figure 1). This body of work is extremely valuable in examining the changing role of central banking in financialisation (for example, see Braun and Gabor, 2020; Fontan and Larue, 2021; Walter and Wansleben, 2020). However, this literature rarely considers spatial dimensions. On the other hand, there is a growing literature at the intersection of *financialisation studies* and *spatial theories* that specifically considers spatial dimensions of financialisation (for example, see Aalbers, 2008; Christophers, 2012; Fernandez and Aalbers, 2020; French et al., 2011; Pike and Pollard, 2010; Sokol, 2013, 2017). However, this literature—with few exceptions (see below)—has so far paid only limited attention to monetary issues.

As a consequence of this, there is still a significant gap at the very epicentre of these debates, at the joint intersection of all three fields (Figure 1). Valuable contributions in filling this gap have been made, for example, by Lapavitsas et al. (2010), Rossi (2013), Celi et al. (2018), Kaltenbrunner and Painceira (2018) or Fernandez and Aalbers (2020) (see also Sokol and Pataccini, 2020). However, most of these contributions focus on either global or national scales—for example, highlighting the impact on the Global South of monetary interventions made in the Global North (for example, Fernandez and Aalbers, 2020; Kaltenbrunner and Painceira, 2018) or describing the impacts of monetary unions (such as the eurozone) on constituent national economies (for example, see Celi et al., 2018; Lapavitsas et al., 2010; Lapavitsas et al., 2012; Rossi, 2013; see also Vermeiren, 2017). Works dealing with spatial impacts on *regional* (sub-national) scales of financialised monetary policy, however, are hard to find (but see Fingleton et al., 2015; Martin, 2001a; Rodríguez-Fuentes and Dow, 2003; Rodríguez-Fuentes, 2006). The need to fill this gap has only increased with the changing role of central banks in the context of financialisation (and recent crises), examined in turn.

## Central banking and monetary policies in the age of financialisation

Standard textbooks on banking (Casu et al., 2015, 123) describe monetary policy as being ‘concerned with the actions taken by central banks to influence the availability and cost of money and credit by controlling some measure (or measures) of the money supply and/or the level and structure of interest rates’. In short, central banks manipulate the amount and the price of money in the economy. Traditionally, the most powerful tool that central banks had for doing this was to set the interest rate (essentially setting the price at which they lend monies to private sector banks, in the hope that this will translate into size and price of credit in the wider economy). As beautifully summed up by Lanchester (2010, 172), central bankers' job ‘is to notice everything and think about everything—everything economic—and then to act on it via one tool and one tool only: the interest rate’. However, as Lanchester notes, interest rate is ‘a fairly crude tool: it’s as if the central banker were sitting at a desk console with thousands of flashing lights and digital readouts and heads-up visual displays, all pouring in overwhelming quantities of data, and in response to it the banker can move only one lever, in straight line backward or forward, and preferably only a very little at a time’ (Lanchester, 2010, 173). This is of course an oversimplification as central banks do much more than this, but under normal circumstances, ‘the interest rate is the whole ball game’ (Lanchester, 2010, 172, note).

Importantly, even small changes in the interest rate have significant implications for the economy; they determine or influence ‘the level of borrowing, the level of credit, the level of economic activity, the level of inflation, the level of unemployment, the speed of growth, the exchange rate, the whole kaboodle’ (Lanchester, 2010, 172–173). Interest rate changes also have significant distributional effects within the economy—both socially and spatially (with the two dimensions being closely intertwined). In terms of social effects, the Fed’s interest rate policy, for example, has been ‘vital to both financial profits and to profits accruing to the capitalist class in general’ (Lapavitsas and Mendieta-Muñoz, 2016; see also Epstein, 2019a). In spatial terms, it is clear that centrally-set interest rates have differentiated effects on the constituent parts of a monetary union as, for instance, highlighted by Mann (2010) through the example of Canadian regions: with the Bank of Canada's monetary policy firmly based on aggregate national data, the eventual effects of the policy may be ‘leaving more political-economically and spatially remote regions like the Atlantic provinces further and further behind’ (Mann, 2010, 613). Of particular concern are differentiated regional impacts on wages and unemployment, with central banks routinely favouring stronger regions at the expense of weaker ones, in the interest of the national economy. In the UK, for instance, the Governor of the Bank of England once infamously suggested that ‘unemployment in the North of the UK is “a price worth paying” for keeping national inflation low’ (quoted in Martin, 2001a, 56, note 4). All these issues are even more contentious within monetary unions such as the eurozone, where entire countries can be affected (see Section 4).

Meanwhile, we would like to underline the fact that the significance of interest rate setting has only increased with the onset of financialisation. Indeed, from the late 1970s and early 1980s onwards, economic policy in advanced capitalist countries has become preoccupied with inflation, and monetary policy has become central to controlling it. In fact, for some, the control of inflation is now part of the very definition of a central bank (for example, see Casu et al., 2015, 122). Price stability (low and stable inflation) has, over the last few decades, increasingly become ‘the most important goal of monetary policy’ (Mishkin et al., 2013, 275). This is based on a ‘broad consensus’ that ‘price stability is an essential pre-condition for achieving the central economic objective of high and stable levels of growth and employment’ (Casu et al., 2015, 126) and monetary policy is seen as ‘the preferred policy choice for influencing prices’ (ibid). It is as if—out of those ‘thousands of flashing lights and digital readouts’ alluded to by Lanchester—only one matters: inflation.

But it is important to note that the rate of inflation is not neutral in its impact on different economic actors. Indeed, high inflation is ‘bad for creditors’ (for example, banks) because it ‘erodes the real value of the loans’ (Jackson, 2020b). The opposite is true for indebted households, firms or governments (see also Lanchester, 2010, 179–180). An important concern for capitalists is, of course, the extent to which inflation can translate into labour wage inflation, thus reducing profits (see also Lapavitsas and Mendieta-Muñoz, 2016).

The anti-inflation drive and the related technique of ‘inflation targeting’ (for example, see Mann, 2010) have, since the late 1980s, gone hand in hand with the mantra of ‘central bank independence’ (Fontan and Larue, 2021). As a result of this, central banks—essentially ‘the government authorities in charge of monetary policy’ (Mishkin et al., 2013, 275)—have become ‘politically independent’ (Fontan and Larue, 2021, 156) from the government. This move was driven by the assumption that ‘politicians were fiscally irresponsible and thus needed independent central banks to bring them into line’ (Tooze, 2020a). All this marked a turning point for central banks and monetary policy. Indeed, it has been noted that, historically, ‘monetary policy has, to a certain extent, been subservient to fiscal and other policies’ involved in macro-economic management, ‘but nowadays it can be regarded as *the main policy tool* used to achieve … economic policy objectives’ (Casu et al., 2015, 127; emphasis added).

Clearly then, the emergence of central banks as seemingly independent technocratic institutions has further strengthened their power. It has also further cemented their role in fostering financialisation. Indeed, the fact that central banks are now independent from democratically elected governments does not mean that they are also independent from other forces such as financial markets—quite the opposite (Fontan and Larue, 2021, 159). In fact, it appears that entanglements between financial markets and central banks have become stronger than ever: central banks are now dependent on financial markets for the very operation of their monetary policies (Braun, 2020b; Braun and Gabor, 2020), while financial markets are dependent on central bank operations for their functioning (Walter and Wansleben, 2020). This mutual interdependence seems to have accelerated the process of financialisation, with monetary policy becoming ‘a constitutive part of financialized capitalism’ (Walter and Wansleben, 2020, 646) and with central banks—‘the elephant in the room of financialization’, acting as ‘decisive catalysts for the crucial development at the heart of financialization’, namely the rise of shadow banking (Braun and Gabor, 2020, 242). Shadow banking has, in turn, contributed a great deal to the increased leverage and excessive debt of the early 2000s (Fernandez and Wigger, 2016). In short, central banks in the Western capitalist world have played a central role in promoting financialisation and the accompanying shift in gravity away from the productive economy and towards the financial sphere (see also Krippner, 2011; Lapavitsas and Mendieta-Muñoz, 2016). As Lapavitsas and Mendieta-Muñoz (2016) argue, central banks have been ‘a pivot of financialisation’ in both high-income and middle-income economies (see also Braun, 2020a; Gabor, 2011). In the context of the European Union (EU), it has been argued that the European Central Bank ‘has emerged as protector of financial interests and guarantor of financialisation in the eurozone’ (Lapavitsas et al., 2012, 3).

There are two major problems with this kind of central banking: increasing inequality (both social and spatial) and increasing financial instability, all of which are interrelated. With regard to inequality, it is important to underline that central bank policies have clearly had distributional effects (Fontan and Larue, 2021), effectively shifting the balance of power between labour and capital in favour of the latter (for example, see Jackson, 2019; Lapavitsas and Mendieta-Muñoz, 2016). In the US context, where the Fed has a dual (and potentially conflicting) mandate of promoting both price stability *and* full employment, this also meant that taming inflation was prioritised over safeguarding workers' jobs (Jackson, 2019). Job losses, in turn, always have their geographies (in that sense social effects can be spatial), thus further exacerbating territorial inequalities between booming and declining regions. Indeed, as Tooze (2020a) has observed, the Fed's ‘sky-high interest rates’ under Paul Volcker plunged ‘Americas’ industrial heartland into crisis’ while also managing to permanently weaken organised labour. In many ways, the economic effects of Volcker's 1980 ‘savage interest rate hike on the steelworkers of the Rust Belt’ (Tooze, 2020a) are still felt today.

With regard to the financial instability issue, it is clear in retrospect that the post-1980s monetary regime helped to create a financialised economic model that was unsustainable. This fully manifested itself in 2007/2008 with the onset of the Global Financial Crisis (GFC). Central bank policies, for example fuelling the credit boom by keeping interest rates too low for too long, are frequently cited as a major contributing factor to the subsequent bust (for example, Lanchester, 2010). However, the involvement of central banks in this financialisation-induced crisis clearly runs much deeper. Remarkably, the mutual interdependence between central banks and financial markets—instead of being disrupted—was further strengthened by the crisis.

Indeed, colossal interventions by the leading central banks prevented a systemic failure: financial markets and the ‘real economy’ would have collapsed completely without robust central bank actions. These included ultra-low interest rates and massive injections of liquidity via Quantitative Easing (QE) programmes. The Fed led the way by injecting some $4.4 trillion into financial markets via its QE programmes between 2009 and 2014 alone, with another $2.8 trillion added by the ECB and yet more by the Bank of England and the Bank of Japan (Jackson, 2019; Tooze, 2018). These interventions show just how central the central banks have become to the survival of contemporary capitalist economies. Yet, it is important to note that these interventions only added to the growing mountain of debt,^1^ thus making the whole economic system even more vulnerable to any further crisis (Sokol and Pataccini, 2020, 410) and thus also more dependent on future central bank actions. Meanwhile, with interest rates already close to zero, central banks have run out of their traditional ammunition and have become even more dependent on financial markets for the transmission of their increasingly unconventional monetary policy.

**Figure 2.**
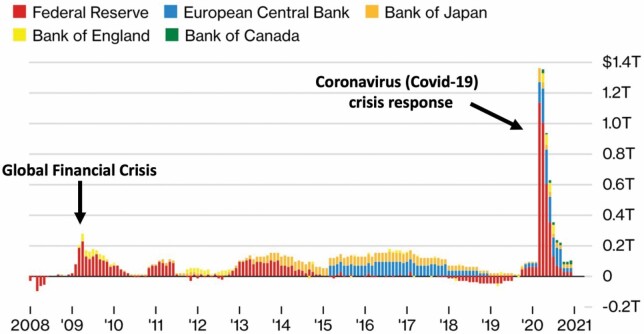
Quantitative Easing (QE) 2008–2021. Source: Adapted from Bloomberg Economics in Boesler and Takeo, 2020.

The resilience of the post-GFC system has been tested by the 2020 pandemic. The coronavirus-induced crisis has further highlighted and strengthened the role of central banks as saviours of financialised capitalism (see Sokol and Pataccini, 2020). Indeed, gargantuan interventions by central banks around the world (Cavallino and De Fiore, 2020) have, yet again, prevented a total financial and economic meltdown (for example, Jackson, 2020a, 2020b; Tooze, 2020b). The scale of these interventions has been unprecedented, dwarfing the previous rounds of QE (Figure 2). At one stage, the Fed was pumping about $1 million into the financial system *every second* (Jackson, 2020b). In terms of crisis governance, Jackson (2020a) remarks, ‘the United States is not a country with a central bank’ rather ‘it is a central bank with a country’.

The breathtaking size of central bank operations (some of them without upper limit) has been combined with an expanded array of monetary interventions. In the Fed's case it looks like these interventions amount to ‘QE infinity’ with the US central bank becoming ‘the ultimate buyer of last resort’ (MacKenzie, 2020). The crisis has greatly enhanced the toolbox of central banks around the world and further expansion is possible (see Section 5). Key to the current efforts by central banks is massive purchases of sovereign debt, that is, government bonds (Gabor 2021), which are essential for the functioning of financial markets (Gabor and Ban, 2016; Gabor and Vestergaard, 2018). Simultaneously, these purchases are also reducing the cost of borrowing for governments (Gabor, 2021, 22), thus giving fiscal authorities some valuable breathing space. Under these circumstances, the fiscal policies of governments are in effect fully dependent on the monetary actions of central banks. It is no exaggeration to say that, as a consequence of these developments, central banks have become the most powerful economic policy-making institutions in contemporary financialised capitalism (see also Braun, 2020a; Tooze, 2020a, for similar arguments).

**Figure 3.**
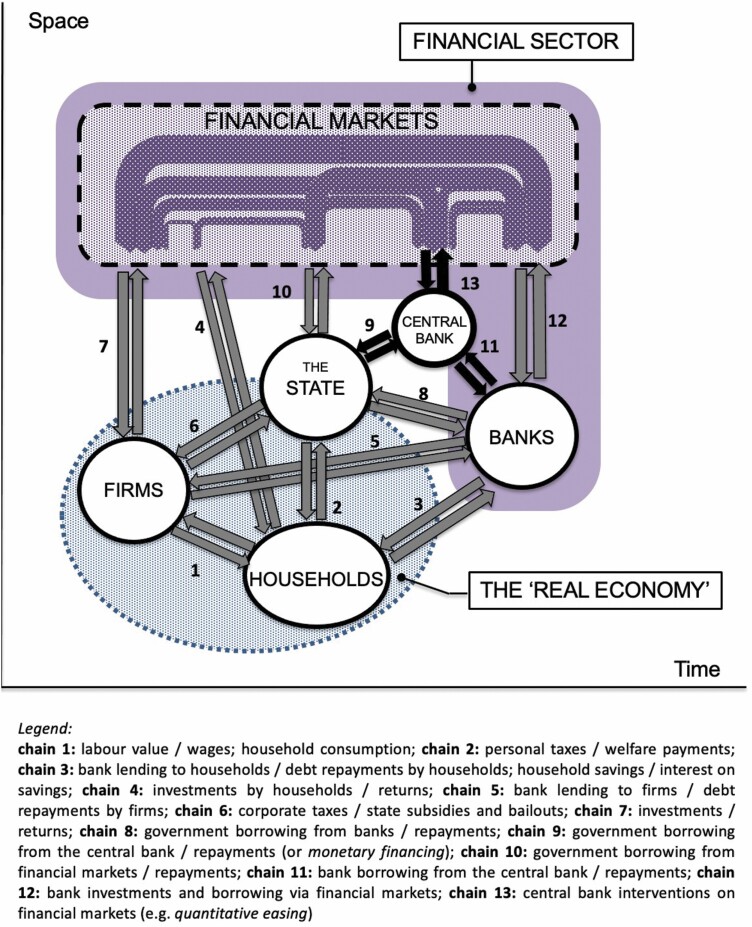
Financial chains in a financialised economy. Source: Adapted from Sokol (2013, 508); Sokol and Pataccini (2020, 405).

In order to better illustrate the position of central banks in the contemporary financialised economy, we employ a ‘financial chains’ perspective (Sokol, 2017; Sokol and Pataccini, 2020), which provides a simplified ‘model’ of financial interactions between key economic players and highlights the central position occupied by the central bank (Figure 3). Of major importance is financial chain No 13 linking the central bank with financial markets and which includes the provision of liquidity via QE. As Tooze (2020a) has observed, liquidity provision is ‘the slogan under which central banks now backstop the entire financial system on a near-permanent basis’. Crucially, central banks were about the only institutions capable of saving the entire economic system when the pandemic hit (Sokol and Pataccini, 2020).

However, the point we wish to emphasise here is that the above ‘financial chains’ model needs to be seen in a time-space dimension. Indeed, all financial flows depicted in Figure 3 are unfolding in time and over space. Monetary actions (that is, the financial flows emanating from the central bank) make their impacts in concrete geographies (places) and over particular timescales. We suggest that the gargantuan interventions implemented by the central banks during the pandemic crisis are likely to make the spatial implications of monetary policies even more pronounced—even though, at this point, we cannot be certain of their exact shape or the concrete timeframes at which these will manifest themselves. What we do know is that these interventions are happening precisely at a moment when spatial divides in general, and regional disparities in particular, are likely to deepen (Sokol and Pataccini, 2020). The issue is of critical importance to monetary unions such as the eurozone, whose space-monetary challenges will be examined in turn.

## The ECB and space-monetary challenges of the eurozone

The European Central Bank is ‘the second-most important central bank in the world’ (Tooze, 2020a) and, according to Mishkin et al. (2013, 290), ‘the most independent central bank in the world’. Yet, the ECB is also one of the youngest central banks in the world. Established under the Treaty on European Union (the Maastricht Treaty), it came to full light in January 1999 with the introduction of the euro as the single currency^2^ for the participating countries of the Economic and Monetary Union (EMU) (Mishkin et al., 2013, 286). As such, the ECB is also, in many ways, a unique central bank with a unique set of (space-)monetary challenges.

One of the unique features of the ECB is the fact that it does not have a country: the state is missing (cf. Figure 3). Indeed, there is no European central state to speak of, despite decades of political integration among European nations. In this sense, the European integration project is incomplete and asymmetric, with the monetary arrangements curiously running well ahead of fiscal and political arrangements. Thus, unlike any other central bank, the ECB does not have a government (or Treasury) to liaise with (and so financial chain No 9 depicted in Figure 3 is missing too). This also raises the issue of legitimacy and representativeness (for example, Clark, 2015). The absence of a central European state also means that there are no sovereign ‘EU-government’ bonds for the ECB to sell or buy, and neither is there an EU-wide fiscal policy to complement the ECB’s monetary actions (as fiscal policies remain the domain of national governments, albeit under strict EMU fiscal rules that are not helping the periphery). Finally, there is no pan-European automatic fiscal stabilisation mechanism, which, as discussed earlier, is considered one of the pre-conditions for an optimum currency area^3^ (see also Fingleton et al., 2015; Martin, 2001a). All this creates significant challenges for the operation of the ECB and contributes to space-monetary tensions.

Further challenges stem from a geographical mismatch between the eurozone and the European Union. Indeed, as of 2021, only 19 countries (of 27 EU countries) have joined the euro area.^4^ This means that the ECB's monetary policy is not automatically transmitted over the entire territory of the European Union. On the other hand, there are countries outside the eurozone, or indeed outside the EU, that unilaterally use the euro as a currency. What we have here, therefore, is a complex monetary space.

This complexity creates challenges—for example with regard to cross-border banking (and lending): in some cases, banks based in eurozone countries operate subsidiaries in non-eurozone countries, while in other cases, banks from non-eurozone countries have subsidiaries in eurozone countries. Furthermore, the transmission of the ECB's monetary policy can be challenging *within* the eurozone itself. Indeed, it is well known that, from the outset, the eurozone has been marked by a major economic divide between the ‘core’ (Austria, Belgium, Finland, Germany, Luxembourg and the Netherlands) and the ‘periphery’ (Greece, Ireland, Italy, Portugal and Spain), with France in between (for example, see Gräbner et al., 2020)—far from ideal as an optimum currency area. Subsequent expansions of the eurozone beyond this original group of 12 countries^5^ only added to its already significant heterogeneity,^6^ with the level of diversity even higher at the regional level (for example, see Fingleton et al., 2015).

The resilience of this diverse monetary union was tested to the limit by the Global Financial Crisis of 2008, and the economic-geographical diversity (especially the imbalance between the northern ‘core’ and the southern ‘periphery’) clearly played a major role in the ensuing ‘Euro crisis’ (for example, see Celi et al., 2018; Christopherson et al., 2015; Gräbner et al., 2020; Hadjimichalis, 2011; Lapavitsas et al., 2010). What is important to note here is that the developments in the core and periphery are structurally interdependent (Overbeek, 2012; Stockhammer and Kohler, 2020) and that ECB monetary policies seem to have exacerbated internal eurozone imbalances by fuelling debt-driven growth in the periphery (with the credit from the core), thus deepening the conditions for the crisis (for example, Gräbner et al., 2020, 657–659; Lapavitsas et al., 2010, 350–356; Rossi, 2013). Moreover, the crisis only served to further deepen the core-periphery divides, both at the level of national economies (Celi et al., 2018; Gräbner et al., 2020) and at the regional economic level (Fingleton et al. 2015), with parts of the eurozone running ‘the risk of perpetual crisis’ (Christopherson et al., 2015, 846).

Apart from slashing its interest rate, the response from the ECB to the crisis (following much earlier moves by the Fed and the Bank of England) was to eventually unleash a QE programme in 2015 (see Figure 2), in the hope that the additional liquidity would further reduce interest rates and make borrowing cheaper.^7^ The expected Europe-wide economic recovery has never fully materialised (for example, see Gräbner et al., 2020) but the ECB's whatever-it-takes actions did help to temporarily stabilise the eurozone. Through these crisis interventions, the ECB greatly enhanced its position as the most important economic policy-making body in Europe while also highlighting the spatial-monetary tensions within the eurozone.

The recent German Constitutional Court's ruling on the ECB's 2015 QE programme (Bodoni, 2020; Sandbu, 2020a) only serves to underline the matter. Indeed, the key complaint is precisely that the ECB has overstepped its mandate by venturing from its narrow ‘monetary policy’ remit into the realm of ‘economic policy’ (see Tooze, 2020a for a detailed discussion). Leaving aside the legal controversy, the German judges also stated the obvious: the monetary policy decisions made by the ECB at the *European* level has economic policy effects for *national* economies in the eurozone—just as the Bank of England’s policy decisions taken at the national level would have for regional economies in the UK (for example, see Dow and Montagnoli, 2007). Spatial tensions caused by monetary interventions are hard to dispute (see also Rodríguez-Fuentes and Dow, 2003; Tuori, 2016). Our point is that the spatial implications of central bank actions will become even more prominent in the wake of the pandemic-induced crisis.

## Policy options and the coronavirus crisis: towards spatial monetary policy?

It could be argued that the Covid-19 pandemic has opened a new chapter in central banking and monetary policy. Central banks have been ‘single-handedly saving the day’ yet again, while becoming ‘more powerful that ever’ (Tooze, 2020a). However, because of this, ‘pandemic central banking’ (Lane, 2020) has also attracted increasing interest from critical scholars and opened up a much-needed debate on the role of central banks in financialised capitalism. Echoing calls made by a growing number of scholars (for example, Braun, 2020a; Braun et al., 2020; Fontan and Larue, 2021; Mazzucato et al., 2020; Ryan-Collins, 2020; Tooze, 2020a), we argue that the crisis presents an opportunity to challenge the established policy paradigms and to reconsider the remit of central banks. Indeed, if we accept the premise that central banks are now key economic policy-making institutions, we need to make sure that their mission is aligned with societal needs and the challenges we are facing. Our key point here is that, in addition to economic, social and environmental concerns, the new remit of central banks must also include spatial considerations.

We suggest that, in the case of the ECB, this would mean that its mandate is fully realigned with the EU objectives of social cohesion (including employment), environmental sustainability (low-carbon economy) and, importantly, territorial cohesion. In pursuing these objectives, the ECB should be willing to deploy creative unconventional approaches (see below). Importantly, the spatial dimensions of any monetary intervention should be carefully considered in order to support territorial cohesion. One way or another, a progressive agenda for regional development in Europe and elsewhere must recognise the central bank as the most important economic policy-making institution, and monetary policy as a key element of spatial policy. Conversely, monetary policy should incorporate balanced territorial development as one of its goals. In the case of the eurozone, embracing an explicit spatial agenda could be seen as a matter of self-preservation: growing spatial polarisation could tear the monetary union apart. What the eurozone needs is a ‘spatial monetary policy’, working in concert with reinvigorated fiscal policies.

There are a number of unconventional tools that could potentially be mobilised to achieve the above objectives. Importantly, such tools need to recognise the extraordinary power that central banks now have, but use it to fulfil positive societal aims. This means that, rather than providing unlimited support for financial markets and banks, monetary policy could directly support households, firms and/or governments. A direct monetary support for governments is a hallmark of the Modern Monetary Theory (MMT)^8^ that is currently gaining traction in unorthodox economic circles (for example, Kelton, 2020; Mitchell et al., 2019; Wray, 2020). At the heart of the MMT is a recognition that a sovereign central bank can, technically, create an unlimited supply of (electronic) money and pass it on to the Government/Treasury for spending—a process that is sometimes referred to as ‘monetary financing’ (see also Diessner, 2020). In effect, this is financial chain No 9 in Figure 3, but without the need to pay back. Indeed, there is nothing to repay—*pure* monetary financing is not a loan: new money is created, but without corresponding debt.^9^

The attractiveness of such an approach is clear. In the case of the eurozone/EU, this could provide breathing space for already heavily indebted peripheral countries (provided that legal hurdles for the ECB to do this are overcome). Such monetary financing would be a radical departure from the ECB’s current approach which only encourages increased sovereign indebtedness through ultra-low (or even negative) interest rates. The opponents of the MMT (for example, Buiter, 2020; Issing, 2020; Rogoff, 2020) most frequently evoke the spectre of the ensuing inflation as a key problem. However, it appears that in the post-pandemic world it is a devastating *deflation* that we need to worry about (Jackson, 2020b; see also Galbraith, 2021; Stiglitz, 2021). Monetary financing, therefore, would make macro-economic sense, while at the same time it could support a range of social goals (full employment), environmental measures (green transition) measures and, we would add, spatial objectives (balanced territorial development). An MMT-style monetary policy, of course, cannot achieve these goals on its own—but it could provide the necessary funds for fiscal authorities to act, blurring the fiscal/monetary policy boundaries in the process.^10^

A slightly different version of monetary financing is for a central bank to create (electronic) money and pass it on directly to either firms or households—so-called *helicopter money* (Diessner, 2020; Fontan and Larue, 2021; Jourdan, 2020; Sandbu, 2020b). Helicopter money for households is sometimes also referred to as *People’s QE* or *QE for people* (for example, Jackson, 2020b; Lonergan and Jourdan, 2016). As evident from Figure 3, currently, there is no direct link between the central bank and either firms or households. Helicoptering money directly to firms or citizens would therefore represent a far-reaching innovation in monetary policy, further blurring the distinction between monetary and fiscal policies. In the eurozone, such a ‘(quasi-)fiscal’ (Diessner, 2020, 9) monetary financing intervention may be a way around the problem of the absence of a central European state and the lack of common fiscal policy. The relative independence of the ECB may be an advantage here—the bank may be better placed than any of its peers to introduce innovative monetary policies and to pioneer new tools. It seems that the ECB could implement some of these within its current legal mandate (Diessner, 2020; Lonergan and Jourdan, 2016), although a ‘new monetary constitution’ (Tooze, 2020a) involving a radical re-think of central banks’ remit may be preferable.

There is a range of other innovative interventions that may be available to the ECB and other central banks (for example, see Fontan and Larue, 2021; Lonergan, 2020; Lonergan and Greene, 2020). However, the point we want to emphasise is that any such policy interventions will be incomplete without considering their spatial impacts. For instance, instead of blanket, spatially blind money drops, central banks may need to come up with carefully calibrated, territorially minded or spatially-targeted helicopter money, as part of their ‘spatial monetary’ policy. Also, the idea of ‘dual interest rates’ (Lonergan and Greene, 2020) could be implemented differently in different regions of the eurozone, in line with their regional needs. In addition, bonds to finance investment in less favoured regions could be issued by a development bank (in the EU this could be the European Investment Bank) and bought by the central bank (the ECB).^11^ There may be further policy options with strong spatial element that could (and should) be explored.

## Conclusion

In this paper, we have highlighted the fact that central banks have become the most powerful economic policy-making bodies in crisis-ridden financialised capitalism. Further, we have argued that, hand in hand with the increasing power of central banks, the spatial effects of monetary interventions are likely to become more significant. The economic fallout of the 2020 pandemic will exacerbate existing spatial inequalities at all geographical scales, while the spatial implications of monetary policies will become even more visible. The enormous power of central banks with regard to spatial development requires urgent attention: what central banks do, and how they do it, matters a lot for uneven geographical development. We thus argue that a progressive agenda for regional economic development must recognise the monetary actions of central banks as a key tool in supporting territorially balanced development. Simultaneously (and alongside economic, social and environmental concerns), monetary policy must embrace spatial considerations. In other words, ‘spatial monetary policy’ is needed, operating in coordination with fiscal and other policies. Echoing calls by Martin (2001b) for a ‘policy turn’ in geography, we suggest that exploring the options for ‘spatial monetary policy’ provides a crucial opportunity for economic and financial geographers to make a difference in the real world.

## Footnotes

1 It is worth noting that not all forms of debt (public/private/domestically owned/foreign owned) inevitably increase the risk of crisis. But it is well known that for weaker economies debt can become unsustainable, with a vicious debt spiral engulfing both public and private debt.

2 First as an ‘invisible’ currency and from January 2002 as cash.

3 The issue is compounded by relatively low labour mobility within the EU.

4
https://www.ecb.europa.eu/euro/intro/html/index.en.html.

5 Eleven countries formed the eurozone in 1999 with Greece joining two years later in 2001.

6 Slovenia joined the eurozone in 2007, followed by Cyprus and Malta (in 2008), Slovakia (2009), Estonia (2011), Latvia (2014) and Lithuania (2015).

7
https://www.ecb.europa.eu/explainers/show-me/html/app_infographic.en.html.

8 See Epstein (2019b) for a detailed account of the emergence of MMT, its theoretical origins and a ‘policy critique’.

9 In a similar way, the UK government has availed of its ‘Ways and Means’ facility at the Bank of England to draw down funds to fight the pandemic in 2020, but here the money is (at least formally) expected to be repaid at some stage (see https://www.bankofengland.co.uk/news/2020/april/hmt-and-boe-announce-temporary-extension-to-ways-and-means-facility).

10 It is important to note that all currencies (including the euro) are part of the international hierarchy of money (currently dominated by the US dollar). Any MMT-style intervention will thus affect, and be affected by, the international political economy (see also Epstein, 2019b). It is therefore likely that some sort of international (global) arrangement would need to be put in place to facilitate the process of direct monetary financing.

11 We thank one of our reviewers for highlighting this particular option.
